# Multi-Institutional Drug Use Patterns in Hospitalized Older Patients: Retrospective Cross-Sectional Study

**DOI:** 10.2196/78353

**Published:** 2026-01-29

**Authors:** Chung Chun Lee, Grace Juyun Kim, Suhyun Kim, Jee Young Hong, Won Min Hwang, Jong-Yeup Kim, Kye Hwa Lee, Kwangsoo Kim, Mingyu Kang, Ju Han Kim, Suehyun Lee

**Affiliations:** 1 Department of Biomedical Informatics College of Medicine Konyang University Daejeon Republic of Korea; 2 Asan Institute for Life Sciences Asan Medical Center Seoul Republic of Korea; 3 Department of Transdisciplinary Medicine, Institute of Convergence Medicine with Innovative Technology Seoul National University Hospital Seoul Republic of Korea; 4 Department of Clinical Medical Sciences College of Medicine Seoul National University Seoul Republic of Korea; 5 Department of Preventive Medicine College of Medicine Jeonbuk National University Jeonju Republic of Korea; 6 Division of Nephrology, Department of Internal Medicine College of Medicine Konyang University Daejeon Republic of Korea; 7 Healthcare Data Science Center Konyang University Hospital Daejeon Republic of Korea; 8 Department of Information Medicine Asan Medical Center Seoul Republic of Korea; 9 Department of Biomedical Informatics College of Medicine University of Ulsan Ulsan Republic of Korea; 10 Department of Medicine College of Medicine Seoul National University Seoul Republic of Korea; 11 Department of Internal Medicine College of Medicine Chungbuk National University Cheongju Republic of Korea; 12 Seoul National University Biomedical Informatics (SNUBI), Division of Biomedical Informatics College of Medicine Seoul National University Seoul Republic of Korea; 13 College of IT Convergence Gachon University Seongnam-si, Gyeonggi-do Republic of Korea

**Keywords:** polypharmacy, older patients, medication, electronic health record, Common Data Model

## Abstract

**Background:**

A rapidly aging population led to an increase in the number of patients with chronic diseases and polypharmacy. Although investigations on the appropriate number of drugs for older patients have been conducted, there is a shortage of studies on polypharmacy criteria in older inpatients from multiple institutions.

**Objective:**

The aim of this study was to examine the patterns of polypharmacy and determine the criteria for the number of drugs defining polypharmacy in the geriatric inpatient population.

**Methods:**

Electronic health records of 4 medical institutions for patients aged 65 years and older hospitalized between January 1, 2012, and December 31, 2020, were analyzed for the study. The maximum number of drugs prescribed was obtained for each patient and, along with a literature review, was used to determine the appropriate polypharmacy level for our population.

**Results:**

We suggest a 4-level polypharmacy category system consisting of nonpolypharmacy, polypharmacy, major polypharmacy, and excessive polypharmacy based on a review of international guidelines and polypharmacy literature. Application of this system to our study population showed that the major polypharmacy category (use of 10-19 concurrent drugs) was an appropriate threshold for polypharmacy in hospitalized patients versus the traditional threshold of 5 or more concurrent drugs. The tendency of our study population to have a higher disease and drug count supports this threshold. Frequently prescribed therapeutic subgroups in this category were antibacterials for systemic use, anesthetics, and cardiac therapy.

**Conclusions:**

This study proposes a polypharmacy categorization system for older inpatients, which differs from the common definition of the concomitant prescription of 5 or more drugs. The older population tends to have severe conditions including those requiring major surgeries; therefore, a drug count corresponding to the definition of major polypharmacy is appropriate.

## Introduction

South Korea is reported to have the highest rate of aging among the Organisation for Economic Cooperation and Development countries, where 8.2 of 51.8 (15.7%) million people in 2020 were aged 65 years or older [1]. The prevalence of chronic diseases in this population was found to be 8481 of 10,097 (84%) individuals aged 65 years or older, making it highly likely that the cost of health care is increasing [2]. This requires close review and consideration of effective measures to prevent and treat chronic diseases and adverse events from polypharmacy.

With the increase in lifespan and aging population in South Korea, patients commonly have multiple concomitant diseases. Consequently, patients visit 1 or more health care institutions and are prescribed medications that expose them to polypharmacy [3]. In 2006, Intercontinental Marketing Statistics Korea found that an average of 4.16 medications were prescribed per health care appointment, which was the highest among the 10 countries studied [2]. A study using the South Korean health claims data of 2016-2019 reported that in 2016, a total of 1548 of 47,631 (3.3%) individuals aged 45 years or older experienced polypharmacy, defined as the use of 10 or more medications for at least 60 days, and this rate increased gradually up to 2019, where 2012 of 48,249 (4.2%) individuals aged 45 years or older were on polypharmacy [4]. To ensure that older adult patients receive appropriate drug therapy, the Korean Health Insurance Review & Assessment Service launched the Drug Utilization Review program. This provides real-time assessment of inappropriate patient drug therapy, including the use of duplicative drug ingredients or drugs of different therapeutic classes, inappropriate drugs for older adults, or drugs not to be used in combination [5].

Considering that older adults have decreased physiological functions and often have several chronic diseases, they are at a higher risk of polypharmacy and hospitalization. The use of at least 5-6 drugs is a definition widely used for polypharmacy in the ambulatory population, and several studies report 25% or more older adults on polypharmacy [2,6-9]. However, this definition may not be practical for the hospitalized older adult population, as this demographic tends to receive more treatments for multiple diseases. Previously reported definitions of polypharmacy in the geriatric hospitalized population were the prescription of 5-9 medications and that of hyper-polypharmacy were 10 or more medications [10,11]. There were also cases of using 5 or more drugs as the definition of polypharmacy for hospitalized patients [12,13]. Although definitions of polypharmacy applicable to older hospitalized patients exist, most studies use single-institution prescription data, making the findings difficult to generalize. Studies from multiple institutions are needed to determine and apply definitions of polypharmacy in the geriatric inpatient population.

Previous studies have used various data sources and methods to define polypharmacy. Data sources included health insurance claims, electronic medical records, spontaneous adverse drug event (ADE) reports, and surveys. The methods used to define polypharmacy include simple numeric definitions, numeric definitions with terms of duration in a therapy or health care setting, and nonnumeric descriptive definitions [14]. Definitions of polypharmacy are diverse due to differing patient age, underlying disease, and disease severity. Accordingly, it is necessary to review the existing definitions of polypharmacy to understand the categories used in various clinical settings and to apply the most appropriate method when assessing the status of polypharmacy in the study population.

Kim et al [2] reported that approximately 6.30 million patients aged 65 years or older in South Korea were prescribed drugs for at least 90 days during 2018, and among this population, 2.64 (41.9%) million were prescribed 5 or more drugs, and 0.77 (13.9%) million were prescribed 10 or more drugs. Cresswell et al [15] reported an increased risk of ADEs with a higher number of prescribed drugs. The older adults were especially at risk of serious long-term outcomes, including death and disability, from these ADEs [15]. Ensuring safer therapy for this population is important. Hanlon et al [16] found that in 167 adults aged 65 years and older, ADEs occurred in 58 (35%), and the most frequent ADEs were gastrointestinal disorders. This was followed by ADEs of the nervous, respiratory, endocrine, skin, and subcutaneous systems [16]. Among the 58 patients who had an ADE, 49 (84%) patients required medical care: 37 (63%) were examined by a physician, 7 (11%) were hospitalized, and 5 (10%) visited the emergency room [16]. Mabuchi et al [17] compared the number of drugs used in patients with a spontaneous ADE report to the overall patient population in Japan using real-world data. In both populations, the number of drugs used increased with age [17].

This study used anonymized electronic health record (EHR) data converted to the Observational Medical Outcomes Partnership (OMOP) Common Data Model (CDM) format, a standardized data structure that accommodates retrospective observational studies [18]. The OMOP CDM data content is generated by extracting EHR data recorded during patient care, removing personal identifiable information, transforming the data into a standardized structure, and mapping hospital-specific terms to standardized, controlled vocabularies. Each CDM in the 4 institutions included patient demographic, condition, measurement, procedure, and drug prescription information [19]. Overall, we examined the characteristics and patterns of polypharmacy in older hospitalized adults across 4 health care institutions (presented as medical institutions A-D) and created a polypharmacy categorization system for this population. Specifically, for each polypharmacy category, we characterized the drugs and drug classes prescribed to the population of interest and examined their drug use patterns.

## Methods

### Study Participants and Observation Period

EHR data of 4 medical institutions converted to the OMOP CDM format were used to analyze polypharmacy patterns. The study participants were patients aged 20 years or older hospitalized at one of these 4 institutions between January 1, 2012, and December 31, 2020. The number of patients aged 20 years or older who visited the medical institutions in the study period ranged from 494,875 to 3,349,795. The number of hospitalized patients, hospitalization events, hospitalized patients aged 60 years and older, and the number of drug classes prescribed to the overall hospitalized patients are presented in Table 1.

**Figure 1:**
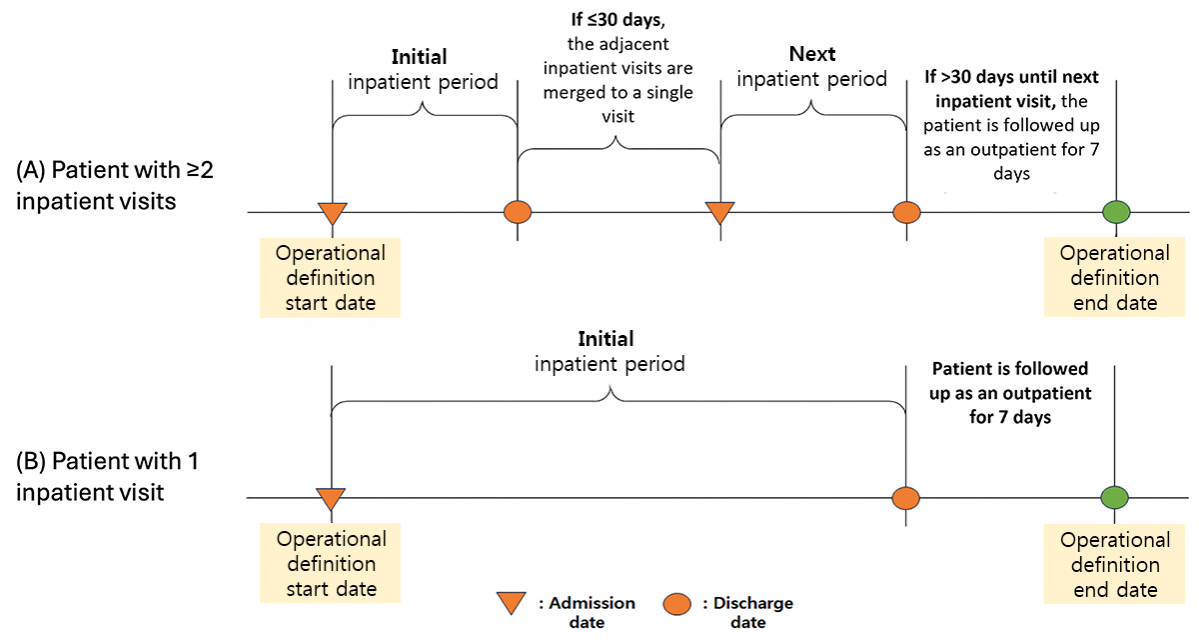
Definition of patient observation period.

To extract study participants, we consulted clinicians from medical departments who frequently encountered polypharmacy. The patient visit types were restricted to inpatients. The earliest date of patient admission was the patient study entry date; if the discharge date of this admission and the admission date of the next admission were within 30 days, the 2 admissions were treated as a single continuous admission period. To investigate the status of discharge drug use, drug exposure data from the outpatient setting were followed up for 7 days after patient discharge. For example, if the patient had 2 admission episodes, the total admission period would be the sum of the duration of admission 1 in days, 7 days after discharge from admission 1, the duration of admission 2 in days, and 7 days after discharge from admission 2 (Figure 1).

**Table 1:** Patient characteristics by medical institution.

	Medical institution A	Medical institution B	Medical institution C	Medical institution D
Patients, n (%)	602,483 (100)	1,944,140 (100)	3,349,795 (100)	494,875 (100)
Hospitalized patients, n (%)	169,931 (20.2)	405,956 (20.9)	419,460 (12.5)	128,700 (26)
Hospitalization events, n (%)	315,624 (100)	1,092,936 (100)	871,764 (100)	212,489 (100)
Older hospitalized adults aged ≥60 years, n (%)	48,512 (8.1)	166,071 (8.5)	113,573 (3.4)	33,997 (6.9)
Drug therapeutic subgroups (WHO^a^ ATC^b^ second-level codes [20]) prescribed, distinct n (%)	79 (84)	86 (91.5)	72 (76.6)	81 (81.2)

^a^WHO: World Health Organization.

^b^ATC: Anatomical Therapeutic Chemical.

### Polypharmacy Level

This study examined the distribution of patients by polypharmacy level for each medical institution. The polypharmacy levels increase by the number of concomitant drugs prescribed to the patient. The cutoff values for concomitant drug counts in each polypharmacy level were determined based on internationally recommended thresholds and existing literature on polypharmacy in older adults. The World Health Organization (WHO) and several countries defined the use of 5 or more drugs as polypharmacy [1,14]. WHO differentiates between appropriate and inappropriate polypharmacy by defining appropriate polypharmacy as evidence-based, personalized medication prescribing and inappropriate polypharmacy as irrational, excessive prescribing [21]. It emphasizes the importance of reducing inappropriate polypharmacy by continuously ensuring the appropriate, rational use of medications [21]. The European Geriatric Medicine Society states that polypharmacy patients should be closely managed through medication reviews to prevent inappropriate polypharmacy [22]. The National Institute for Health and Care Excellence recommends monitoring the benefits and harms of each drug taken by adults using 15 or more regular medications [23].

Masnoon et al [14] used concurrent drug counts, admission duration, and treatment duration to define polypharmacy, and Young et al [24] determined the category of polypharmacy by the number of concomitant drugs and the presence of high-risk drugs in the patient’s drug regimen. Studies on hospitalized, older patients used polypharmacy levels other than the widespread definition of 5 or more drugs [25-27]. An investigation of the physical performance of hospitalized older patients defined polypharmacy as the use of 10 or more drugs during hospital stay [26]. A study analyzing risk factors of increased hospital stay in older adults defined polypharmacy as the concomitant use of 5 to 9 drugs and excessive polypharmacy as 10 or more drugs [25]. The polypharmacy definition for nursing home patients as well as older populations in general was also 5-9 concurrent drugs, and that of excessive polypharmacy was 10 or more concurrent drugs [27-29]. Kim et al [30] divided polypharmacy into 3 levels: concomitant use of 6 or more drugs as polypharmacy, 11 or more drugs as major polypharmacy, and 21 or more drugs as excessive polypharmacy [30].

Based on WHO recommendations and the literature search mentioned earlier, we suggested a polypharmacy categorization method to detect regimens, including an excessive number of drugs, with an aim to assist in detecting inappropriate polypharmacy (Table 2). Polypharmacy was defined as concomitant prescription of 5 or more drugs, which is consistent with the widespread definition [14] and based on previous studies on polypharmacy in older adults. The next severe polypharmacy level, which was major polypharmacy, was defined as the prescription of 10-19 drugs. This threshold was used, as it was commonly used in the older patient population [25-29]. The most severe polypharmacy level, excessive polypharmacy, was defined as the prescription of 20 or more drugs based on a previous study of polypharmacy use among older adults [30].

**Table 2:** Definition of polypharmacy based on the number of concomitant drugs.

Concomitant drugs, n	Polypharmacy category
<5	Nonpolypharmacy
5-9	Polypharmacy
10-19	Major polypharmacy
≥20	Excessive polypharmacy

### Data Preprocessing and Data Analysis Environment

Preprocessing of the medical institution data was performed by converting the EHR to the OMOP CDM (version 5.3.1) format. We used the following inpatient data extracted from the CDM tables: person, drug_exposure, and visit_occurrence. The data for each CDM table were extracted for the years 2012-2020. Using drug_exposure OMOP CDM data, the severity of polypharmacy and drug use patterns in the patients were analyzed. Based on clinical professional consultation, the study patients were divided into those with 1 admission and those with 2 or more admissions. The operational definition of continuous admission was applied to the data of patients with multiple admissions within 30 days to obtain the final admission counts. Drug exposure data in the outpatient setting for 7 days after follow-up were extracted for each patient. We extracted the drug exposure data for all patients during their observation period and for each day of drug exposure and counted the number of drug ingredients exposed to the patient. The date of the maximum number of dispensed drug ingredients was determined. If the patient was exposed to this maximum number of drug ingredients for multiple days, the date closest to admission was used as the date of the maximum drug ingredient count. The drug ingredient count on that date was used to determine the patient’s polypharmacy level. To perform the drug ingredient mapping process, we excluded patients without at least 7 days of drug exposure data after discharge.

Data processing and analyses were performed using R (version 4.1.3; R Foundation for Statistical Computing). Specifically, CDM data access, extraction, and conversion into variables were performed using R packages *RpostgreSql* (version 0.7-8) [31] and *sqldf* (version 0.4-11) [32]. R packages used for data preprocessing were *dplyr* (version 0.8.3) [33] and *data.table* (version 1.12.2) [34], and that used for descriptive analyses was *moonBook* (version 0.3.1) [35]. The *ggplot2* (version 3.2.1) [36] R package was used for data visualization. Medical institute A used 16 GB RAM in the Windows operating system (OS), medical institutes B and C used 64 GB RAM in the Windows OS, and medical institute D used 128 GB RAM in the Windows OS.

### Polypharmacy Pattern Analysis

For each patient, we mapped the WHO Anatomical Therapeutic Chemical (ATC) code to each drug exposure event in the OMOP CDM. Medical institutions A, C, and D assigned WHO ATC codes to drugs in the OMOP CDM using electronic data interchange (EDI) codes. This is possible because the EDI codes are loaded into the institutional CDM. Mapping the WHO ATC codes to EDI codes required a file with code information for drugs distributed in South Korea provided by the Korea Pharmaceutical Information Service [37]. Korea Pharmaceutical Information Service, operated by the Health Insurance Review & Assessment Service, assigns WHO ATC codes to drugs approved in South Korea. The code information file includes EDI, WHO ATC, and drug ingredient codes for each drug approved; therefore, assigning WHO ATC and drug ingredients to each drug in the OMOP CDM was possible. Traditional Korean medications and drugs without WHO-ATC, EDI, and drug ingredient codes were excluded from the mapping process.

Medical institution B did not have EDI codes available in the OMOP CDM; therefore, a different approach was used to assign WHO ATC codes to the drug concept IDs. This institution maps the WHO ATC code to each drug in the CDM drug_exposure table using the concept and concept_ancestor tables. If the drug_concept_id in the drug_exposure table was a descendant_concept_id of an ancestor_concept_id indicating a fifth-level WHO ATC code, then the concept_id and WHO ATC codes were mapped to the drug_concept_id. The concept table was used to determine if the ancestor_concept_id was a WHO ATC code, and the concept_ancestor table was used to determine if drug_concept_id was a descendant_concept_id of a WHO ATC code.

The maximum daily drug ingredient count was used to determine the polypharmacy level. A maximum drug ingredient count of less than 5 drugs was considered nonpolypharmacy, 5-9 drug ingredients as polypharmacy, 10-19 drug ingredients as major polypharmacy, and 20 drug ingredients or more as excessive polypharmacy.

Demographic characteristics were analyzed using the processed CDM data. The characteristics examined included the total number of inpatients and their admissions, patient age, precautionary drug use, proportion of older adults, admission duration, and drug ingredient exposure distribution for the years 2012-2020.

**Figure 2:**
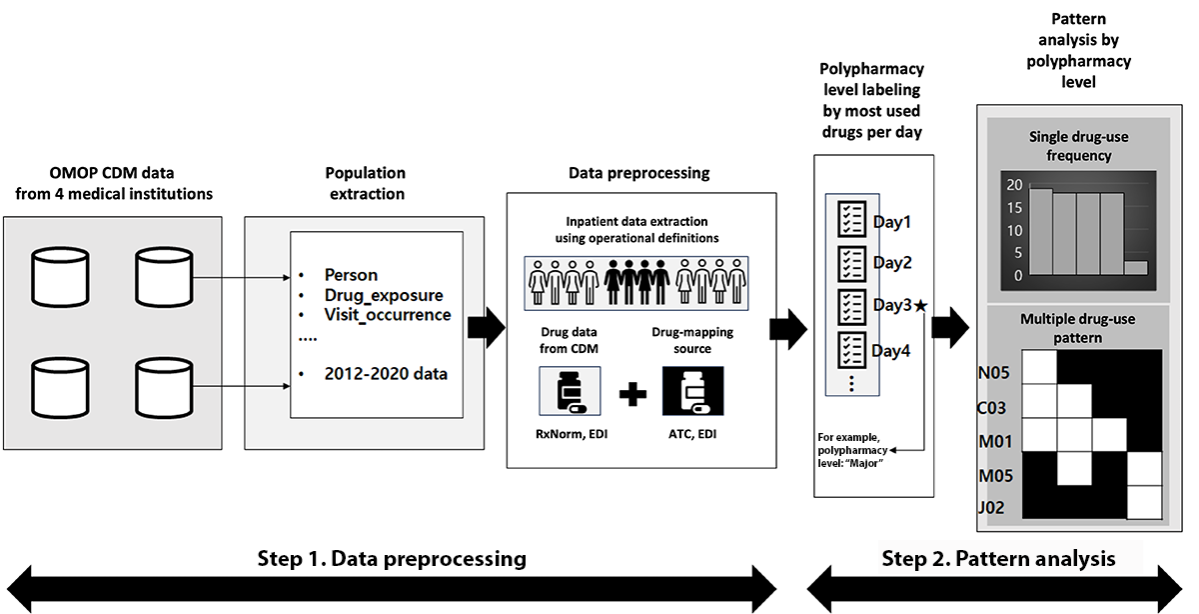
Workflow for polypharmacy pattern determination. ATC: Anatomical Therapeutic Chemical; C03: diuretics; CDM: Common Data Model; EDI: electronic data interchange; J02: antimycotics for systemic use; M01: anti-inflammatories and antirheumatics; M05: drugs for treatment of bone diseases; N05: psycholeptics; OMOP: Observational Medical Outcomes Partnership.

We performed single and combination drug class pattern analyses using the second ATC level as the unit for the drug class. This level was selected, as it represents clinically meaningful therapeutic groups, allowing the comparison of prescribing trends across multiple health care institutions. We excluded drugs in the ATC second-level B05 (blood substitutes and perfusion solutions) for the analyses, as these drugs have a low risk of interacting with other drugs, have little clinical significance, and are used in the majority of inpatients. [38]. The drug use patterns were visualized using the *ggplot2* R package. Single-drug class use patterns were analyzed by determining the number of patients using each drug class at least once. Combination drug class patterns were analyzed by first determining the date of the highest number of drugs prescribed for each patient and examining the drug classes used on that date. The workflow of the study is depicted in Figure 2.

### Ethical Considerations

The study was conducted in accordance with the Declaration of Helsinki and approved by the institutional review board of Konyang University Hospital (protocol code: KYUH2022-07-015-001, approved on September 21, 2022), Asan Medical Center (protocol code: 2022-0342, approved on October 21, 2022), Seoul National University Hospital (protocol code: E-2209-069-1358, approved on September 20, 2022), and Chungbuk University Hospital (protocol code: CBNUH2022-09-030, approved on October 18, 2022). Patient consent was waived, as the data were collected during the routine care of physicians and removed of personally identifiable information, therefore eliminating the risk of compromising patient privacy. There was no direct contact with any patients during the course of this study, and no compensation was provided to the participants.

## Results

### Polypharmacy Levels and Their Drug Use Patterns

The mean maximum daily drug counts in older adults aged 60-69, 70-79, and ≥80 years were highest in patients admitted to medical institution B followed by medical institution A. Duration of admission increased with higher age range in medical institution C. In all institutions except for institution A, the average hospitalization period of the population aged 70 years or older was longer than the population aged 60-69 years (Table 3).

**Table 3:** Multi-institutional maximum daily number of drugs prescribed and duration of hospitalization in older adults.

Age group (years)	Medical institution A		Medical institution B		Medical institution C		Medical institution D	
	Drugs, mean (SD)	Hospitalization period (days), mean (SD)	Drugs, mean (SD)	Hospitalization period (days), mean (SD)	Drugs, mean (SD)	Hospitalization period (days), mean (SD)	Drugs, mean (SD)	Hospitalization period (days), mean (SD)
60-69	21.3 (10.3)	24.0 (35.5)	24.6 (14.5)	19.3 (25.7)	15.9 (7.0)	17.3 (15.8)	20.0 (10.3)	15.2 (26.3)
70-79	21.5 (10.3)	25.3 (32.4)	25.2 (14.7)	19.7 (24.4)	16.1 (7.1)	18.5 (18.1)	20.3 (10.8)	18.0 (32.7)
≥80	20.9 (9.6)	22.8 (23.5)	24.5 (14.7)	19.3 (28.0)	15.5 (6.9)	19.1 (19.4)	19.8 (10.6)	18.1 (25.7)

### Older Adult Polypharmacy Levels Based on Multi-Institutional CDM Data

The level of polypharmacy with the most patients was major polypharmacy in medical institutions A, C, and D, while for medical institution B, this was excessive polypharmacy (Table 4).

**Table 4:** Distribution of patients by polypharmacy level.

Medical institution and age group (years)			Nonpolypharmacy patients, n (%)		Polypharmacy patients, n (%)		Major polypharmacy patients, n (%)		Excessive polypharmacy patients, n (%)
**Medical institution A**									
	60-69	730 (31.9)		2121 (24.9)		5002 (22.2)		3753 (24.7)	
	70-79	1123 (49.1)		4085 (48)		10,557 (46.9)		7251 (47.6)	
	≥80	436 (19)		2297 (27)		6936 (30.8)		4221 (27.7)	
	Total	2289 (100)		8503 (100)		22,495 (100)		15,225 (100)	
**Medical institution B**									
	60-69	4164 (61.4)		7975 (55.6)		22,847 (53.1)		55,016 (54)	
	70-79	2092 (30.8)		4887 (34.1)		15,422 (35.8)		37,130 (36.4)	
	≥80	528 (7.8)		1486 (10.4)		4756 (11.1)		9768 (9.6)	
	Total	6784 (100)		14,348 (100)		43,025 (100)		101,914 (100)	
**Medical institution C**									
	60-69	3657 (31.9)		8746 (30.9)		18,573 (31.6)		4777 (31.8)	
	70-79	5951 (51.9)		14,634 (51.7)		30,563 (52)		7977 (53.1)	
	≥80	1859 (16.2)		4927 (17.4)		9640 (16.4)		2269 (15.1)	
	Total	11,467 (100)		28,307 (100)		58,776 (100)		15,023 (100)	
**Medical institution D**									
	60-69	963 (26.6)		1954 (24.1)		3018 (22.8)		2173 (24.1)	
	70-79	1909 (52.7)		3991 (49.2)		6448 (48.7)		4609 (51.1)	
	≥80	752 (20.8)		2167 (26.7)		3775 (28.5)		2238 (24.8)	
	Total	3624 (100)		8112 (100)		13,241 (100)		9020 (100)	

### Multi-Institute CDM-Based Polypharmacy Pattern

The pattern of polypharmacy in the older adults was analyzed based on the use of single or combinations of drug therapeutic subgroups (WHO ATC second-level codes). The older population was defined as adults aged 65 years and older, based on the population grouping method of the National Statistical Office of Korea [1].

#### Single Drug Use Patterns

**Figure 3:**
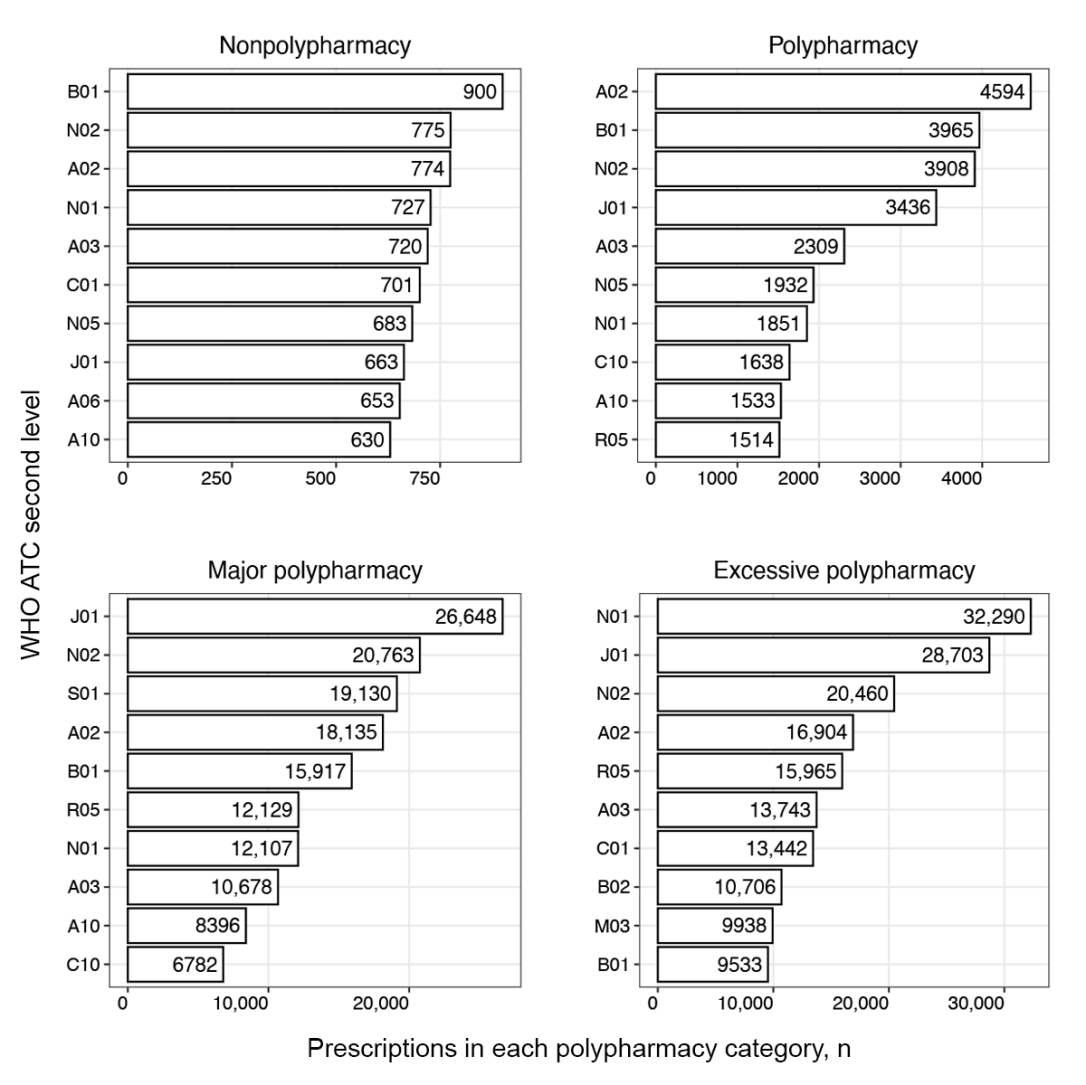
Single therapeutic subgroup (WHO ATC second-level) prescribing pattern in medical institution A. A02: acid-related disorder drug; A03: functional gastrointestinal disorder drug; A06: constipation drug; A10: antidiabetics; ATC: Anatomical Therapeutic Chemical; B01: antithrombotics; B02: antihemorrhagics; C01: cardiac therapy; C10: lipid-modifying agent; J01: systemic antibacterials; M03: muscle relaxant; N01: anesthetics; N02: analgesics; N05: psycholeptics; R05: cough and cold preparation; S01: ophthalmologicals; WHO: World Health Organization.

**Figure 4:**
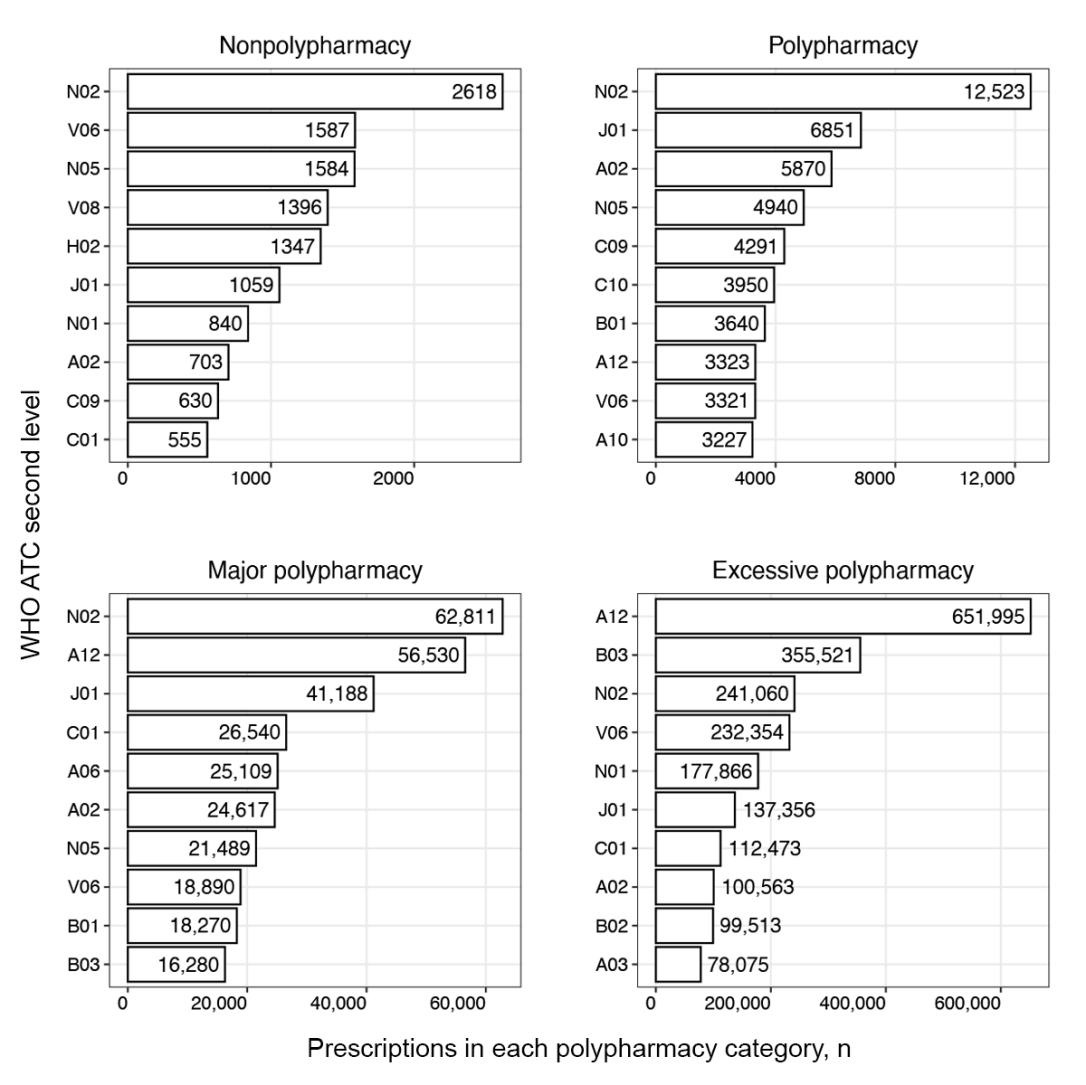
Single therapeutic subgroup (WHO ATC second-level) prescribing pattern in medical institution B. A02: acid-related disorder drug; A03: functional gastrointestinal disorder drug; A06: constipation drug; A10: antidiabetics; A12: mineral supplement; ATC: Anatomical Therapeutic Chemical; B01: antithrombotics; B02: antihemorrhagics; B03: antianemics; C01: cardiac therapy; C09: renin-angiotensin system agent; C10: lipid-modifying agent; H02: systemic corticosteroid; J01: systemic antibacterials; N01: anesthetics; N02: analgesics; N05: psycholeptics; V06: general nutrient; V08: contrast media; WHO: World Health Organization.

**Figure 5:**
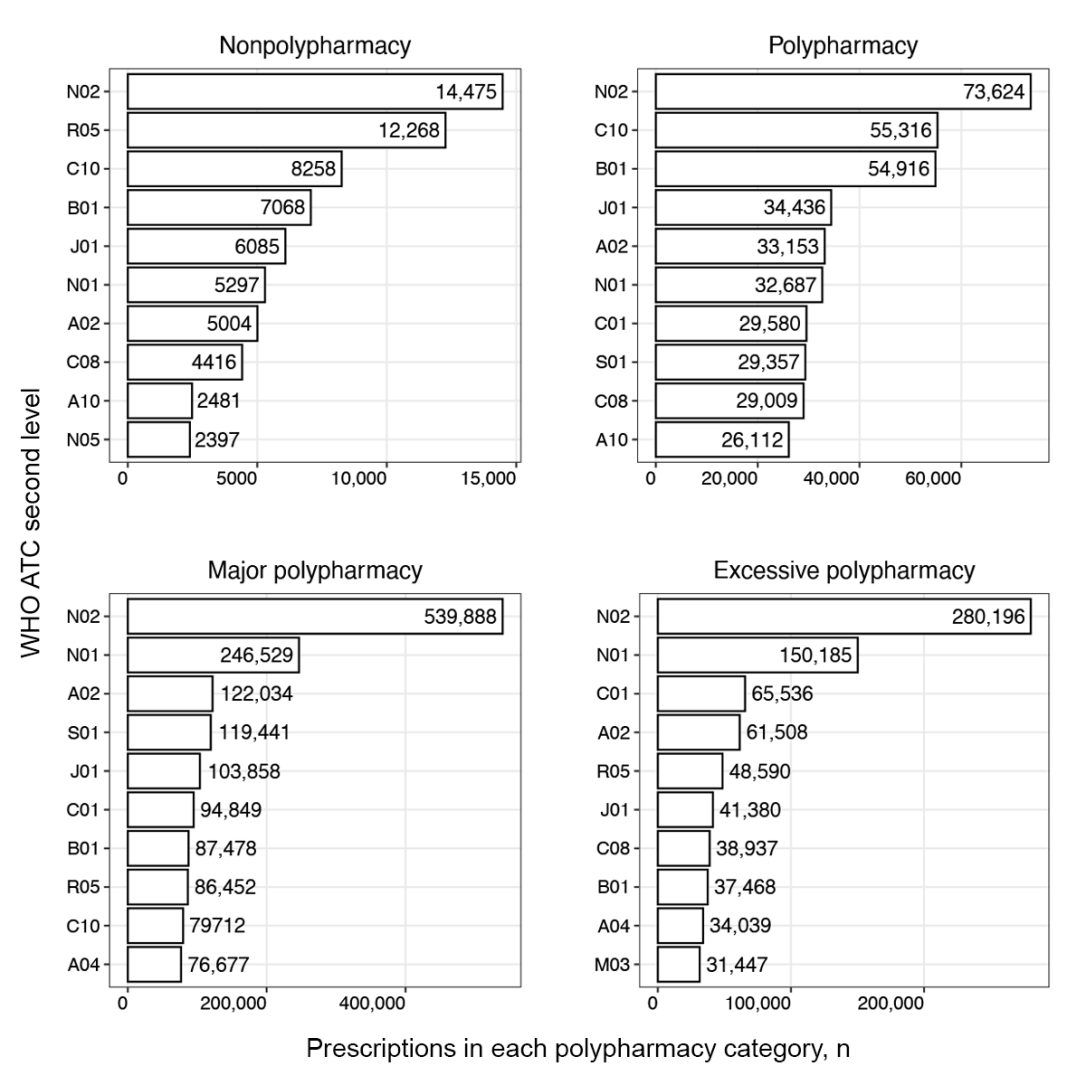
Single therapeutic subgroup (WHO ATC second-level) prescribing pattern in medical institution C. A02: acid-related disorder drug; A04: antiemetics and antinauseants; A10: antidiabetics; ATC: Anatomical Therapeutic Chemical; B01: antithrombotics; C01: cardiac therapy; C08: calcium channel blocker; C10: lipid-modifying agent; J01: systemic antibacterials; M03: muscle relaxant; N01: anesthetics; N02: analgesics; N05: psycholeptics; R05: cough and cold preparation; S01: ophthalmologicals; WHO: World Health Organization.

**Figure 6:**
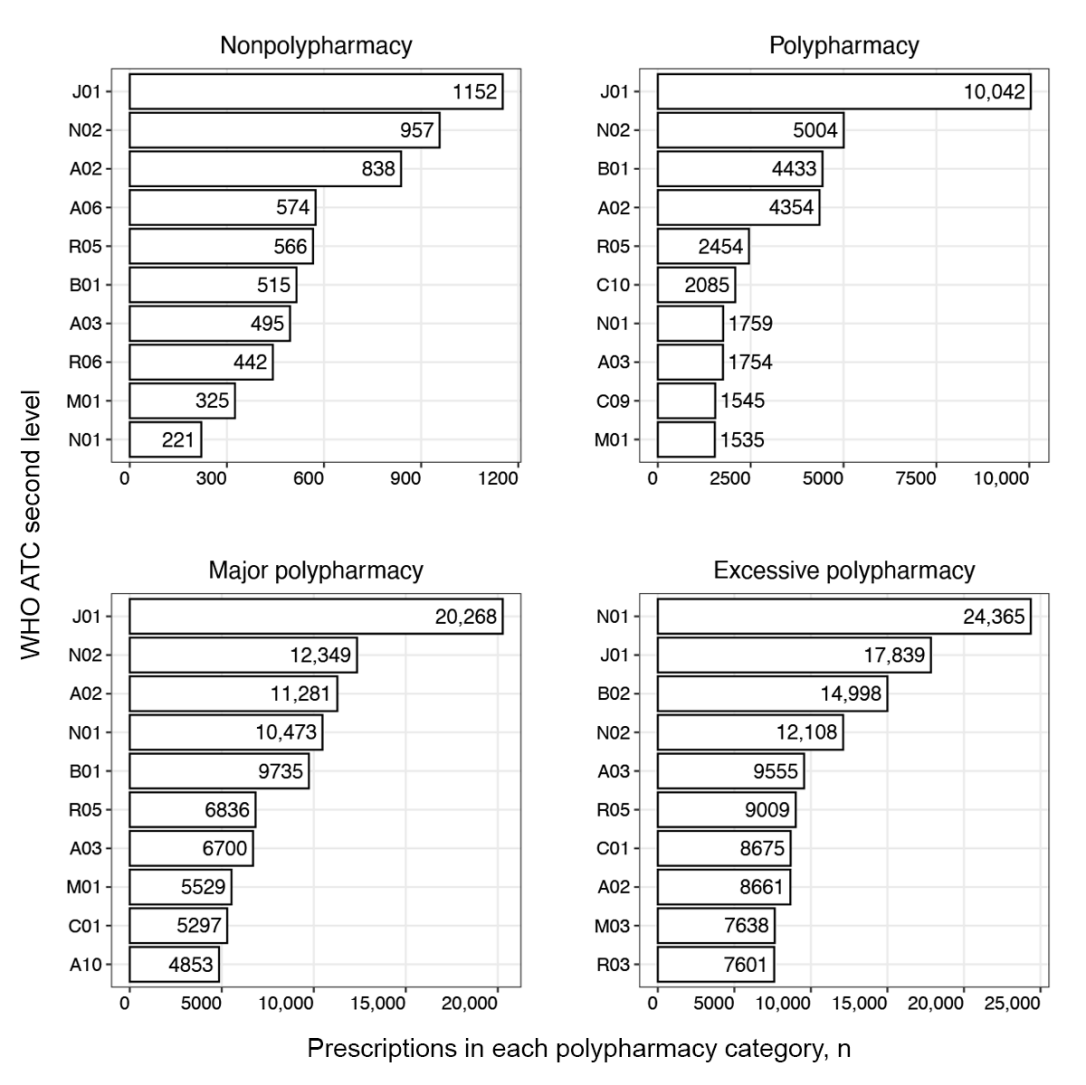
Single therapeutic subgroup (WHO ATC second-level) prescribing pattern in medical institution D. A02: acid-related disorder drug; A03: functional gastrointestinal disorder drug; A06: constipation drug; A10: antidiabetics; ATC: Anatomical Therapeutic Chemical; B01: antithrombotics; B02: antihemorrhagics; C01: cardiac therapy; C09: renin-angiotensin system agent; C10: lipid-modifying agent; J01: systemic antibacterials; M01: anti-inflammatories and antirheumatics; M03: muscle relaxant; N01: anesthetics; N02: analgesics; R03: obstructive airway disease drug; R05: cough and cold preparation; R06: systemic antihistamine; WHO: World Health Organization.

We examined frequently prescribed drug therapeutic subgroups using second-level WHO ATC codes as the subgroup unit. The 10 most frequently prescribed subgroups for each medical institution are presented in Figures 3-6. The horizontal axis represents the number of prescriptions for drugs included in the therapeutic subgroup specified on the vertical axis. According to the 10 most frequently prescribed therapeutic subgroups, the number of prescriptions tended to increase with higher levels of polypharmacy in all medical institutions, excluding institution C. The highest prescription frequency in this institution was observed at the major polypharmacy level.

For medical institution A, the antithrombotics (B01) therapeutic subgroup was frequently prescribed in patients in the nonpolypharmacy and polypharmacy levels compared to other levels and antibacterials for systemic use (J01) for the major and excessive polypharmacy levels. Notably, drugs in the anesthetic (N01) subgroup were most frequently prescribed to patients with excessive polypharmacy (Figure 3).

In medical institution B, patients in all polypharmacy levels except the excessive level were most frequently prescribed analgesics (N02). The most frequently prescribed therapeutic subgroups for patients with excessive polypharmacy included mineral supplements (A12) and antianemic preparations (B03). Patients in all polypharmacy levels were frequently prescribed antibacterial agents for systemic use (J01) and drugs for acid-related disorders (A02). Patients in the major polypharmacy level were frequently prescribed cardiac therapy (C01) and drugs for constipation (A06; Figure 4).

In medical institution C, the analgesic (N02) therapeutic subgroup was prescribed most frequently to patients in all polypharmacy levels. Cough and cold preparations (R05) were relatively frequently prescribed to patients in the nonpolypharmacy level, and lipid-modifying drugs (C10) and antithrombotics (B01) to patients in the polypharmacy level. Anesthetics (N01) were prescribed more frequently to patients in major and excessive polypharmacy levels than to those in other polypharmacy levels (Figure 5).

In medical institution D, antibacterials for systemic use (J01) and analgesics (N02) were prescribed frequently to patients across all polypharmacy levels. Drugs for acid-related disorders (A02) were frequently prescribed to patients in all polypharmacy levels, excluding the excessive level, whereas antihemorrhagics (B02) and anesthetics (N01) were most frequently prescribed to patients in the excessive level (Figure 6).

#### Concomitant Drug Use Patterns

**Figure 7:**
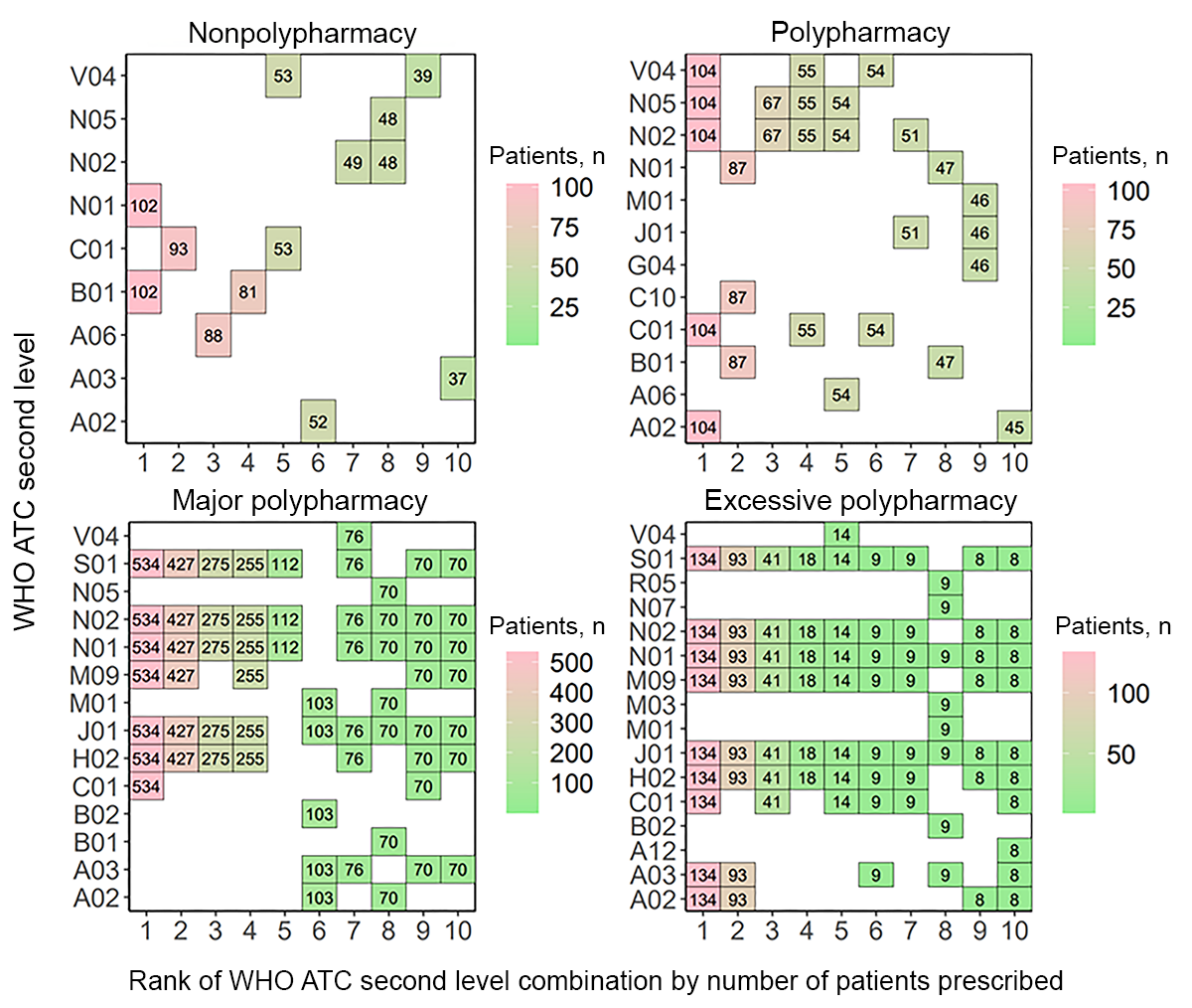
The most frequently prescribed 10 concomitant drug therapeutic subgroups (WHO ATC second-level combinations) used in each polypharmacy level at medical institution A. A02: acid-related disorder drug; A03: functional gastrointestinal disorder drug; A06: constipation drug; A12: mineral supplement; ATC: Anatomical Therapeutic Chemical; B01: antithrombotics; B02: antihemorrhagics; C01: cardiac therapy; C10: lipid-modifying agent; G04: urologicals; H02: systemic corticosteroid; J01: systemic antibacterials; M01: anti-inflammatories and antirheumatics; M03: muscle relaxant; M09: other musculo-skeletal system drug; N01: anesthetics; N02: analgesics; N05: psycholeptics; N07: other nervous system drug; R05: cough and cold preparation; S01: ophthalmologicals; V04: diagnostic agent; WHO: World Health Organization.

Following the examination of single therapeutic subgroup patterns, concomitantly used therapeutic subgroups were analyzed to determine the trends in combination drug use in patients with polypharmacy. At medical institution A, the most frequently prescribed therapeutic subgroup combination for patients of the nonpolypharmacy level was B01 and N01. For patients in the polypharmacy level, A02-C01-N02-N05-V04 was the most frequently prescribed combination. Patients in the major polypharmacy level were most frequently prescribed with the therapeutic subgroup combination C01-H02-J01-M09-N01-N02-S01, while patients in the excessive polypharmacy group were most frequently prescribed the combination A02-A03-C01-H02-J01-M09-N01-N02-S01 (Figure 7). The full names for each of the therapeutic subgroups denoted as WHO ATC second-level codes are in Multimedia Appendix 1.

**Figure 8:**
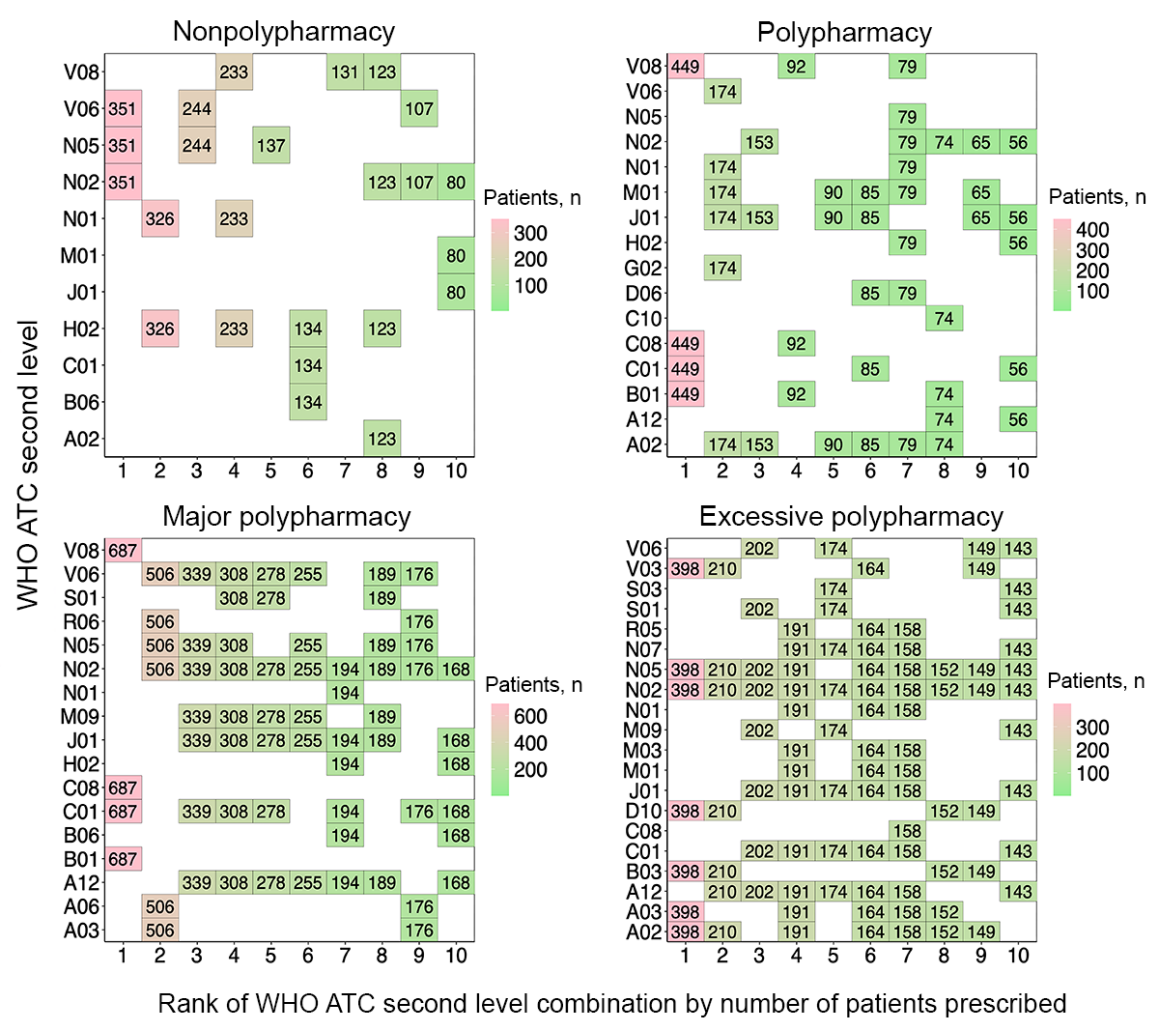
The most frequently prescribed 10 concomitant drug therapeutic subgroups (WHO ATC second-level combinations) used in each polypharmacy level at medical institution B. A02: acid-related disorder drug; A03: functional gastrointestinal disorder drug; A06: constipation drug; A12: mineral supplement; ATC: Anatomical Therapeutic Chemical; B01: antithrombotics; B03: antianemics; B06: other hematological agent; C01: cardiac therapy; C08: calcium channel blocker; C10: lipid-modifying agent; D06: dermatologic antibiotics and chemotherapeutics; D10: antiacne preparation; G02: other gynecologicals; H02: systemic corticosteroid; J01: systemic antibacterials; M01: anti-inflammatories and antirheumatics; M03: muscle relaxant; M09: other musculo-skeletal system drug; N01: anesthetics; N02: analgesics; N05: psycholeptics; N07: other nervous system drug; R05: cough and cold preparation; R06: systemic antihistamine; S01: ophthalmologicals; S03: ophthalmological and otological preparation; V03: other therapeutic product; V06: general nutrient; V08: contrast media; WHO: World Health Organization.

Results for medical institution B showed that patients in the nonpolypharmacy level were most frequently prescribed therapeutic subgroup combination N02-N05-V06, followed by combination H02-N01. The most frequent combination prescribed to patients at the polypharmacy level was B01-C01-C08-V08, followed by A02-G02-J01-M01-N01-V06 and A02-J01-N02. Patients in the major polypharmacy level were also most frequently prescribed the combination B01-C01-C08-V08. The second most frequently prescribed combination at this level was A03-A06-N02-N05-R06-V06, and the third was A12-C01-J01-M09-N02-N05-V06. The most frequently prescribed subgroup combination for patients in the excessive polypharmacy level was A02-A03-B03-D10-N02-N05-V03, followed by A02-A12-B03-D10-N02-N05-V03 and A12-C01-J01-M09-N02-N05-S01-V06 (Figure 8).

**Figure 9:**
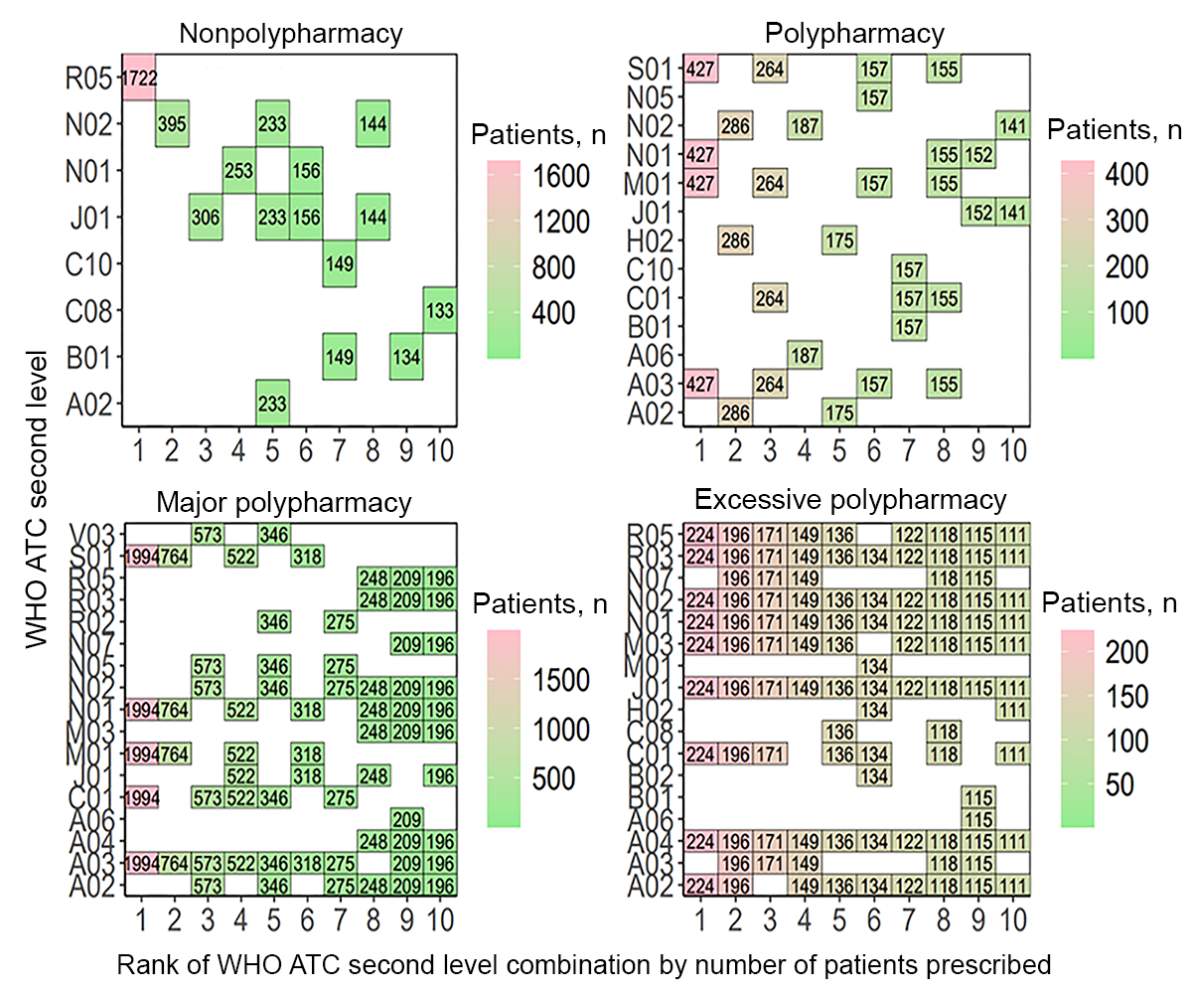
The most frequently prescribed 10 concomitant drug therapeutic subgroups (WHO ATC second-level combinations) used in each polypharmacy level at medical institution C. A02: acid-related disorder drug; A03: functional gastrointestinal disorder drug; A04: antiemetics and antinauseants; A06: constipation drug; ATC: Anatomical Therapeutic Chemical; B01: antithrombotics; B02: antihemorrhagics; C01: cardiac therapy; C08: calcium channel blocker; C10: lipid-modifying agent; H02: systemic corticosteroid; J01: systemic antibacterials; M01: anti-inflammatories and antirheumatics; M03: muscle relaxant; N01: anesthetics; N02: analgesics; N05: psycholeptics; N07: other nervous system drug; R02: throat preparation; R03: obstructive airway disease drug; R05: cough and cold preparation; S01: ophthalmologicals; V03: other therapeutic product; WHO: World Health Organization.

In medical institution C, the 3 most frequent therapeutic subgroup combinations prescribed to patients at the nonpolypharmacy level each involved 1 subgroup: R05, N02, and J01. Patients in the polypharmacy level were most frequently prescribed therapeutic subgroup combination A03-M01-N01-S01. The most frequent subgroup combination prescribed for patients in the major polypharmacy level was A03-C01-M01-N01-S01, followed by A03-M01-N01-S01 and A02-A03-C01-N02-N05-V03. Patients in the excessive polypharmacy level were prescribed A02-A04-C01-J01-M03-N01-N02-R03-R05 most frequently. The second frequent combination was the same as the most frequent combination with the addition of subgroups A03 and A07. The third most frequent combination matched the second, excluding subgroup A02 (Figure 9).

**Figure 10:**
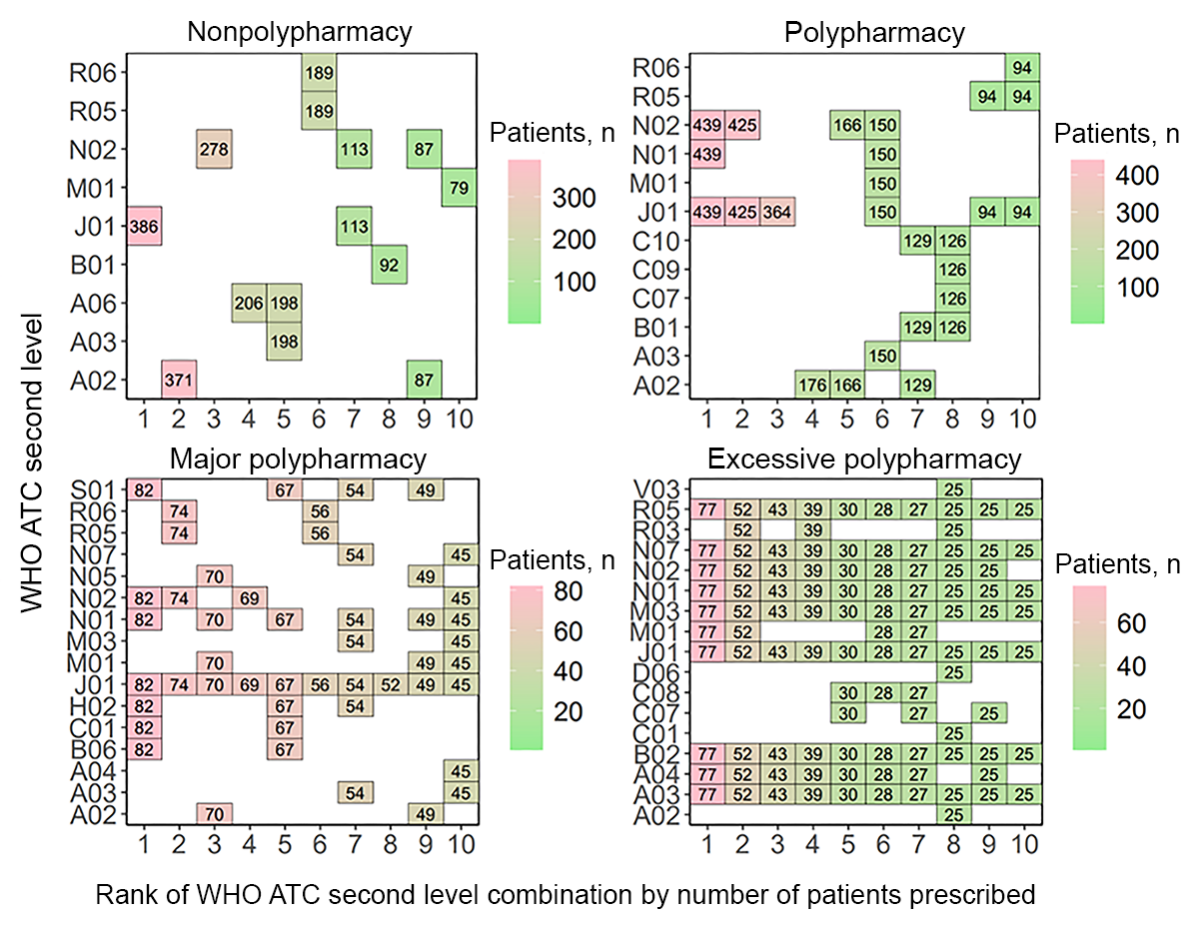
The most frequently prescribed 10 concomitant drug therapeutic subgroups (WHO ATC second-level combinations) used in each polypharmacy level at medical institution D. A02: acid-related disorder drug; A03: functional gastrointestinal disorder drug; A04: antiemetics and antinauseants; A06: constipation drug; ATC: Anatomical Therapeutic Chemical; B01: antithrombotics; B02: antihemorrhagics; B06: other hematological agent; C01: cardiac therapy; C07: beta-blocking agent; C08: calcium channel blocker; C09: renin-angiotensin system agent; C10: lipid-modifying agent; D06: dermatologic antibiotics and chemotherapeutics; H02: systemic corticosteriod; J01: systemic antibacterials; M01: anti-inflammatories and antirheumatics; M03: muscle relaxant; N01: anesthetics; N02: analgesics; N05: psycholeptics; N07: other nervous system drug; R03: obstructive airway disease drug; R05: cough and cold preparation; R06: systemic antihistamine; S01: ophthalmologicals; V03: other therapeutic product; WHO: World Health Organization.

In medical institution D, patients in the nonpolypharmacy level were prescribed therapeutic subgroup J01 most frequently, followed by individual subgroups A02, N02, and A06. Patients in the polypharmacy level were mostly prescribed the combination J01-N01-N02. Those in major polypharmacy level were prescribed B06-C01-H02-J01-N01-N02-S01, followed by J01-N02-R05-R06 and A02-J01-M01-N01-N05. Patients in the excessive polypharmacy level were prescribed combination A03-A04-B02-J01-M01-M03-N01-N02-N07-R05 most frequently, and the second and third most frequent combinations involved the addition of subgroup R03 and the removal of subgroup M01, respectively (Figure 10).

## Discussion

### Principal Findings

This study used EHR data converted to the OMOP CDM from 4 medical institutions for geriatric inpatients aged 65 years and older admitted over 9 years. Our study is the first to collect the EHR of patients and retrospectively study polypharmacy patterns over multiple institutions to determine a polypharmacy threshold in hospitalized older patients. The authors used data from 2 of the largest hospitals in the South Korean metropolitan area and 2 hospitals in the regional area to represent patients from distinct settings.

The use of OMOP CDM in all 4 study settings allowed the use of similar programming languages and software, thereby increasing the efficiency of the study. Although there were differences in the specific columns customized for each institution, the uniformity of the basic OMOP CDM tables and columns across institutions allowed for the distribution of a single analysis code customized for each institution, and the collection of analysis resulted in a standardized format.

We offer a possible definition of polypharmacy in hospitalized older adults. This was obtained by consulting clinical professionals to define the inpatient observation periods and reviewing previous studies [25,26,30] to determine the time point of drug exposure for polypharmacy level determination. We filtered out drugs that did not contribute to adverse events from polypharmacy (eg, WHO ATC code B05 and its lower-level codes), which improved the quality of our data.

The 4 health care institutions (A, B, C, and D) included in this study showed distinct differences in patient scale, proportion of older patients, sex and age distribution, and medication use patterns. Institution C had the largest patient population with 3,349,795 patients and demonstrated high proportions of older patients and excessive polypharmacy levels. Institution B had the next highest patient population with 1,944,140 patients and the highest number of hospitalization events with 1,092,936 events. This trend reflects that institutions located in metropolitan, urban settings had a higher patient population size and hospitalization event count than their rural counterparts. Institution A, despite having a relatively smaller scale with 602,483 patients, was characterized by the highest average number of medications per age group and the longest hospital stays.

In terms of sex distribution, institution A showed higher male ratios in patients aged 5-60 years, but female patients were more prevalent in those aged 70 years and older. Institution C displayed higher female ratios in patients in their 50s and 80s or older, while male patients were more common in the 60- to 70-year age group. Institution D exhibited a unique pattern with higher female ratios in patients aged 80 years and older and higher male ratios in those aged 5-70 years. Overall, the proportion of female patients tended to increase in the older population, with sex ratios varying across institutions and age groups.

A tendency was observed for hospital length of stay to increase with advancing age across all institutions. Regarding polypharmacy prescriptions, institution B demonstrated the highest “excessive polypharmacy level” ratio across all age groups.

### Comparison to Prior Work

The pattern of drug use among inpatients in the 4 medical institutions showed that patients in the major or excessive polypharmacy levels were most prevalent. This trend is consistent with the results of previous studies reporting 5-10 drugs or more as the polypharmacy threshold. A study using nationwide health care claims data found that hospital admission was significantly associated with polypharmacy (use of 5 or more drugs) and hyper-polypharmacy (use of 10 or more drugs) [39]. Another study reported an increase from 5.6 drugs taken per patient on average at admission to 7.6 drugs at discharge [40]. Considering that the number of drugs prescribed tended to increase during hospital admission, the use of 10-19 or more drugs (major polypharmacy or higher) as the threshold for inpatient polypharmacy was considered appropriate. All data were obtained and analyzed within the securely blocked environment of each medical institution.

The mean number of drugs used in medical institution B was higher than that used in other institutions by 3-10 drugs. The prescription frequency of drugs in the cardiac therapy (C01) WHO ATC therapeutic subgroups tended to increase with the severity of polypharmacy in all medical institutions, and the anti-inflammatory and antirheumatic product (M01) therapeutic subgroups increased across all institutions, excluding institution B. These results correspond with those of a study in which hypertension, osteoarthritis, and rheumatoid arthritis were reported to be prevalent in older individuals aged 65 years or older [41].

The patterns of concomitant therapeutic subgroups differed among the medical institutions. In the major polypharmacy category, medical institution A patients were frequently prescribed ophthalmologicals (WHO ATC second-level code S01), along with other therapeutic subgroups. This drug class has been consistently used in most prescribed combinations of therapeutic subgroups. Medical institution B prescribed drugs in various therapeutic categories including acid-related disorders (A02), functional gastrointestinal disorders (A03), antianemic preparations (B03), antithrombotics (B01), and cardiac therapies (C01) to patients in the major and excessive polypharmacy levels. Medical institution C, which had the highest number of study patients, prescribed drugs for WHO ATC second-level functional gastrointestinal disorders (A03), anti-inflammatory and antirheumatic products (M01), and ophthalmologicals (S01). These drug classes were in the 1-3 highest rank of concomitant drugs prescribed.

Distinct polypharmacy patterns in the polypharmacy group were that patients in all 4 institutions were more frequently prescribed lipid-modifying agents (C10), and in 3 institutions, antidiabetics (A10) were more frequently prescribed, compared to other polypharmacy groups (Figures 3-6). This reflects the increased prevalence of chronic diseases in the older population and their demand for multiple medications to treat the disease [42]. Furthermore, the result is consistent with a finding that older patients with diabetes have a high prevalence of polypharmacy, as these patients commonly have multiple concomitant diseases including cardiovascular, renal, and respiratory disorders [43]. The higher prescription frequencies of ophthalmic agents (S01) and anti-inflammatory and analgesic drugs (M01) observed in institution C reflect that this institution is highly specialized in ophthalmology or rheumatology. The major polypharmacy group was frequently prescribed cough and cold preparations (R05) compared to other polypharmacy groups (Figures 3-6). This trend was consistent with results of a previous study, which reported patients on 5 or more drugs having an odds ratio of 3.18 and 6 or more drugs having an odds ratio of 4.57 to receive prescriptions for cough and cold drugs [44].

Distinctive polypharmacy patterns observed in the excessive polypharmacy group were that cardiac drugs (C01) were frequently prescribed in all 4 institutions (Figures 3-6). This observation suggests that patients in the excessive polypharmacy group may have a higher burden of cardiovascular diseases or more severe cardiac comorbidities requiring complex medication regimens [45]. The frequent prescription of cardiac drugs in this group indicates that managing heart-related conditions—such as heart failure, arrhythmia, or ischemic heart disease—contributes substantially to the overall medication load. This reflects that the patients in the excessive polypharmacy group are clinically more fragile, thus requiring multiple medications for symptom control, prevention of complications, and comorbidity management. This group was also frequently prescribed functional gastrointestinal disorder drugs (A03), antihemorrhagics (B02), and muscle relaxants (M03; Figures 7-10). The reason for the high prevalence of antihemorrhagics prescribing indicates frequent surgeries or procedures in this population [46,47]. Muscle relaxants were found to be frequently prescribed in older polypharmacy patients and coprescribed with opioids [48]. This medication was shown to increase the risk of adverse outcomes including all-cause and cardiovascular mortality, warranting enhanced monitoring of drug therapy [48]. Gastrointestinal drugs may be used for the gastric protection caused by polypharmacy or due to the decreased physiological function of the gastrointestinal system [49].

Although each institution had a distinctive pattern, we observed that drugs related to surgeries, including those needed after the operation (eg, WHO ATC second-level codes C01, H02, J01, N01, and N02), were commonly prescribed across institutions (Figures 7-10). This reflects the trend from 2011 to 2015, where 3,146,452 of 8,497,443 (37%) surgeries were performed in patients aged 65 years or older, and the surgeries were for high-risk conditions, such as glaucoma [50]. We observed that older inpatients frequently underwent surgeries and were prescribed several drugs during admission for these procedures. The high frequency of antibacterials (WHO ATC second-level code J01), anesthetics (N01), and analgesics (N02) in all polypharmacy levels in the older population reflects the complex clinical conditions of hospitalized older patients, who often require management of infections, surgeries or procedures, and pain control [51]. This population has a high prevalence of osteoarthritis, fractures, and cancer-related pain, increasing the risk of anesthetic and analgesic use [52].

### Limitations

This study has several limitations. First, we included 4 medical institutions, which restricts the generalizability of the study results to the institutions of interest. Second, drugs without a mapped WHO ATC code were excluded; therefore, the prevalence of polypharmacy might have been underestimated. Third, we analyzed the pattern of polypharmacy using the second-level ATC code; thus, polypharmacy patterns at the ingredient level were not determined. Fourth, although we examined outpatient drug prescriptions up to 7 days after discharge, polypharmacy use in older outpatients was not comprehensively analyzed. This is a topic of interest for future studies. Fifth, we did not assess the appropriateness of polypharmacy based on linking prescribed drugs and their indications. Although an estimate of the drug-indication linkage is possible, the precise linkage is challenging, as the indications for the drugs were not explicitly recorded in the OMOP CDM database. Finally, the tables used for the analyses did not include the note table, which includes clinical results or interpretations in free-text format, and is a potential resource for detecting polypharmacy.

### Conclusions

Based on well-established EHR-based CDM datasets in South Korea, we suggest a polypharmacy definition for older inpatients that is different from the commonly used definition of the concurrent prescription of 5 or more drugs. This is because, based on the severity of the patients’ condition and the treatment they require, which includes major surgeries, the number of concomitant drugs used was higher than the commonly used definition of polypharmacy in the majority of patients in this study. Therefore, we suggest that 10 or more drugs is a more suitable definition of polypharmacy for geriatric inpatients. As the geriatric population increases, evaluating the appropriateness of existing polypharmacy definitions and serious outcomes of polypharmacy in this population, including renal failure, falls, and fractures, is necessary for future studies.

## Appendix

Multimedia Appendix 1 World Health Organization Anatomical Therapeutic Chemical second-level codes and names.

## Abbreviations

ADE: adverse drug event
ATC: Anatomical Therapeutic Chemical
CDM: Common Data Model
EDI: electronic data interchange
EHR: electronic health record
OMOP: Observational Medical Outcomes Partnership
OS: operating system
WHO: World Health Organization

## Data Availability

The datasets generated or analyzed during this study are not publicly available due to privacy protection policies but are available after approval or exemption by the institutional review boards of the study institutions.
