# Supplementary material for: Multi-Institutional Drug Use Patterns in Hospitalized Older Patients: Retrospective Cross-Sectional Study
Source: JMIR Med Inform. 2026 Jan 29;14:e78353. doi: 10.2196/78353 (PMC12902758; doi:10.2196/78353)
Supplement: Multimedia Appendix 1 [file medinform_v14i1e78353_app1.docx]

**Table S1.** World Health Organization Anatomical Therapeutic Chemical second level codes and names [1].

| **WHO^a^ ATC^b^ 2nd level code (therapeutic subgroup)** | **Classification name** |
| --- | --- |
| A01 | STOMATOLOGICAL PREPARATIONS |
| A02 | DRUGS FOR ACID RELATED DISORDERS |
| A03 | DRUGS FOR FUNCTIONAL GASTROINTESTINAL DISORDERS |
| A04 | ANTIEMETICS AND ANTINAUSEANTS |
| A05 | BILE AND LIVER THERAPY |
| A06 | DRUGS FOR CONSTIPATION |
| A07 | ANTIDIARRHEALS, INTESTINAL ANTIINFLAMMATORY/ANTIINFECTIVE AGENTS |
| A08 | ANTIOBESITY PREPARATIONS, EXCL. DIET PRODUCTS |
| A09 | DIGESTIVES, INCL. ENZYMES |
| A10 | DRUGS USED IN DIABETES |
| A11 | VITAMINS |
| A12 | MINERAL SUPPLEMENTS |
| A13 | TONICS |
| A14 | ANABOLIC AGENTS FOR SYSTEMIC USE |
| A15 | APPETITE STIMULANTS |
| A16 | OTHER ALIMENTARY TRACT AND METABOLISM PRODUCTS |
| B01 | ANTITHROMBOTIC AGENTS |
| B02 | ANTIHEMORRHAGICS |
| B03 | ANTIANEMIC PREPARATIONS |
| B05 | BLOOD SUBSTITUTES AND PERFUSION SOLUTIONS |
| B06 | OTHER HEMATOLOGICAL AGENTS |
| C01 | CARDIAC THERAPY |
| C02 | ANTIHYPERTENSIVES |
| C03 | DIURETICS |
| C04 | PERIPHERAL VASODILATORS |
| C05 | VASOPROTECTIVES |
| C07 | BETA BLOCKING AGENTS |
| C08 | CALCIUM CHANNEL BLOCKERS |
| C09 | AGENTS ACTING ON THE RENIN-ANGIOTENSIN SYSTEM |
| C10 | LIPID MODIFYING AGENTS |
| D01 | ANTIFUNGALS FOR DERMATOLOGICAL USE |
| D02 | EMOLLIENTS AND PROTECTIVES |
| D03 | PREPARATIONS FOR TREATMENT OF WOUNDS AND ULCERS |
| D04 | ANTIPRURITICS, INCL. ANTIHISTAMINES, ANESTHETICS, ETC. |
| D05 | ANTIPSORIATICS |
| D06 | ANTIBIOTICS AND CHEMOTHERAPEUTICS FOR DERMATOLOGICAL USE |
| D07 | CORTICOSTEROIDS, DERMATOLOGICAL PREPARATIONS |
| D08 | ANTISEPTICS AND DISINFECTANTS |
| D09 | MEDICATED DRESSINGS |
| D10 | ANTI-ACNE PREPARATIONS |
| D11 | OTHER DERMATOLOGICAL PREPARATIONS |
| G01 | GYNECOLOGICAL ANTIINFECTIVES AND ANTISEPTICS |
| G02 | OTHER GYNECOLOGICALS |
| G03 | SEX HORMONES AND MODULATORS OF THE GENITAL SYSTEM |
| G04 | UROLOGICALS |
| H01 | PITUITARY AND HYPOTHALAMIC HORMONES AND ANALOGUES |
| H02 | CORTICOSTEROIDS FOR SYSTEMIC USE |
| H03 | THYROID THERAPY |
| H04 | PANCREATIC HORMONES |
| H05 | CALCIUM HOMEOSTASIS |
| J01 | ANTIBACTERIALS FOR SYSTEMIC USE |
| J02 | ANTIMYCOTICS FOR SYSTEMIC USE |
| J04 | ANTIMYCOBACTERIALS |
| J05 | ANTIVIRALS FOR SYSTEMIC USE |
| J06 | IMMUNE SERA AND IMMUNOGLOBULINS |
| J07 | VACCINES |
| L01 | ANTINEOPLASTIC AGENTS |
| L02 | ENDOCRINE THERAPY |
| L03 | IMMUNOSTIMULANTS |
| L04 | IMMUNOSUPPRESSANTS |
| M01 | ANTIINFLAMMATORY AND ANTIRHEUMATIC PRODUCTS |
| M02 | TOPICAL PRODUCTS FOR JOINT AND MUSCULAR PAIN |
| M03 | MUSCLE RELAXANTS |
| M04 | ANTIGOUT PREPARATIONS |
| M05 | DRUGS FOR TREATMENT OF BONE DISEASES |
| M09 | OTHER DRUGS FOR DISORDERS OF THE MUSCULO-SKELETAL SYSTEM |
| N01 | ANESTHETICS |
| N02 | ANALGESICS |
| N03 | ANTIEPILEPTICS |
| N04 | ANTI-PARKINSON DRUGS |
| N05 | PSYCHOLEPTICS |
| N06 | PSYCHOANALEPTICS |
| N07 | OTHER NERVOUS SYSTEM DRUGS |
| P01 | ANTIPROTOZOALS |
| P02 | ANTHELMINTICS |
| P03 | ECTOPARASITICIDES, INCL. SCABICIDES, INSECTICIDES AND REPELLENTS |
| R01 | NASAL PREPARATIONS |
| R02 | THROAT PREPARATIONS |
| R03 | DRUGS FOR OBSTRUCTIVE AIRWAY DISEASES |
| R05 | COUGH AND COLD PREPARATIONS |
| R06 | ANTIHISTAMINES FOR SYSTEMIC USE |
| R07 | OTHER RESPIRATORY SYSTEM PRODUCTS |
| S01 | OPHTHALMOLOGICALS |
| S02 | OTOLOGICALS |
| S03 | OPHTHALMOLOGICAL AND OTOLOGICAL PREPARATIONS |
| V01 | ALLERGENS |
| V03 | ALL OTHER THERAPEUTIC PRODUCTS |
| V04 | DIAGNOSTIC AGENTS |
| V06 | GENERAL NUTRIENTS |
| V07 | ALL OTHER NON-THERAPEUTIC PRODUCTS |
| V08 | CONTRAST MEDIA |
| V09 | DIAGNOSTIC RADIOPHARMACEUTICALS |
| V10 | THERAPEUTIC RADIOPHARMACEUTICALS |
| V20 | SURGICAL DRESSINGS |

^a^WHO: World Health Organization.

^b^ATC: Anatomical Therapeutic Chemical.

**Supplementary Materials Reference**

1. Norwegian Institute of Public Health. ATC/DDD Index. WHO Collaborating Centre for Drug Statistics Methodology. 2025. <https://atcddd.fhi.no/atc_ddd_index/>. Accessed 12 Oct 2022.
